# Lost.—Registers

**Published:** 1884-08

**Authors:** 


### Lost.—Registers.

Within the last year, five or six volumes of the DENTAL REGISTER have been borrowed from our files—where they have gone, or who has them, we know not. The missing volumes are 5, 6, 7, 8, and 25. We do not object to loan books provided they are returned in due time, but do not wish them to go and stay so long, as it might be forgotten where they belong. It is an easy matter to forget about such things. If the books can be returned we shall be very thankful, for the first four can not be replaced.

